# Prosthetic Avian Vocal Organ Controlled by a Freely Behaving Bird Based on a Low Dimensional Model of the Biomechanical Periphery

**DOI:** 10.1371/journal.pcbi.1002546

**Published:** 2012-06-28

**Authors:** Ezequiel M. Arneodo, Yonatan Sanz Perl, Franz Goller, Gabriel B. Mindlin

**Affiliations:** 1 Laboratorio de sistemas dinámicos, Departamento de Física, FCEyN, Universidad de Buenos Aires, Buenos Aires, Argentina; 2 Department of Biology, University of Utah, Salt Lake City, Utah, United States of America; Northeastern University, United States of America

## Abstract

Because of the parallels found with human language production and acquisition, birdsong is an ideal animal model to study general mechanisms underlying complex, learned motor behavior. The rich and diverse vocalizations of songbirds emerge as a result of the interaction between a pattern generator in the brain and a highly nontrivial nonlinear periphery. Much of the complexity of this vocal behavior has been understood by studying the physics of the avian vocal organ, particularly the syrinx. A mathematical model describing the complex periphery as a nonlinear dynamical system leads to the conclusion that nontrivial behavior emerges even when the organ is commanded by simple motor instructions: smooth paths in a low dimensional parameter space. An analysis of the model provides insight into which parameters are responsible for generating a rich variety of diverse vocalizations, and what the physiological meaning of these parameters is. By recording the physiological motor instructions elicited by a spontaneously singing muted bird and computing the model on a Digital Signal Processor in real-time, we produce realistic synthetic vocalizations that replace the bird's own auditory feedback. In this way, we build a bio-prosthetic avian vocal organ driven by a freely behaving bird via its physiologically coded motor commands. Since it is based on a low-dimensional nonlinear mathematical model of the peripheral effector, the emulation of the motor behavior requires light computation, in such a way that our bio-prosthetic device can be implemented on a portable platform.

## Author Summary

Brain Machine Interfaces (BMIs) decode motor instructions from neuro-physiological recordings and feed them to bio-mimetic effectors. Many applications achieve high accuracy on a limited number of tasks by applying statistical methods to these data to extract features corresponding to certain motor instructions. We built a bio-prosthetic avian vocal organ. The device is based on a low-dimensional mathematical model that accounts for the dynamics of the bird's vocal organ and robustly relates smooth paths in a physiologically meaningful parameter space to complex sequences of vocalizations. The two physiological motor gestures (sub-syringeal pressure and ventral syringeal muscular activity), are reconstructed from the bird's song, and the model is implemented on a portable Digital Signal Processor to produce synthetic birdsong when driven by a freely behaving bird via the sub-syringeal pressure gesture. This exemplifies the plausibility of a type of synthetic interfacing between the brain and a complex behavior. In this type of devices, the understanding of the bio-mechanics of the periphery is key to identifying a low dimensional physiological signal coding the motor instructions, therefore enabling real-time implementation at a low computational cost.

## Introduction

The complex motor behavior originating the rich vocalizations of adult oscine birds results from the interaction between a central pattern generator (the brain) and a nonlinear biomechanical periphery (the bird's vocal organ) [1], [2]. The fact that this complex behavior is learned, together with the parallels between the physical mechanisms of birdsong and human speech production, make birdsong an ideal model to study how a complex motor behavior is acquired, produced and maintained [3].

In an effort to understand what gives rise to complexity in this behavior, a part of the birdsong community has set focus on the capabilities of the periphery to produce vocalizations owning a diverse set of nontrivial acoustic features [2]. The avian vocal organ, comprised mainly by the respiratory system, the syrinx and the vocal tract, is a highly nonlinear biomechanical device. The complexity of its dynamics leaves traces in the sounds that can be produced in it. In this way, several acoustic features found in vocalizations can be related to nonlinear phenomena occurring in the syrinx [4], [5] or introduced by acoustic interactions between the syrinx and the tract [6]–[9]. In all these cases, the complexity of the behavior does not require a complex motor pattern to drive the vocal organ, but rather simple, smooth gestures.

Through a combination of experimental observations and theoretical analysis, low-dimensional mathematical models have been proposed that account for the physical mechanisms of sound production in the avian vocal organ [10], [11]. In particular, a model based on Titze's proposed flapping mechanism for oscillations in human vocal folds [12] was recently used to synthesize the song of the Zebra finch (*Taeniopygia guttata*) [13], [14]. This model captures the nonlinear dynamics of the folds oscillating to produce sound, in a way that a variety of complex vocalizations are generated by the tuning of parameters related to physiologically observable motor gestures elicited by the bird.

Part of the appeal of counting with this model is the prospect of applying it to the construction of a bio-prosthetic device. In this scenario computation is relatively inexpensive because of the low dimension of the mathematical model. In addition, the physical description of the peripheral effectors led to the identification of a set of smoothly varying parameters that determine the behavior [13]. By recording the physiological activity related to the parameters and feeding it to a device that solves the equations of the model in real-time, vocal behavior can be emulated by a prosthesis controlled by a subject via its motor instructions.

The usual strategy of BCIs and BMIs (Brain Computer Interfaces and Brain Machine Interfaces) is to decode motor commands from recordings of physiological activity in the brain and use this activity to control bio-mimetic devices [15]–[17]. In [17], for instance, multi-electrode recordings of tens to hundreds of neurons in different cortical areas of primates are used to drive a robotic arm. In recent work, Cichocki *et. al.* discuss the perspectives of using electroencephalographic (EEG) recordings to generate noninvasive BCI solutions [16]. In these examples (as well as in many other BCI implementations), the crucial problem is the classification of the features of the large data set which correspond to a determined set of motor tasks. The feature extraction is performed by different techniques which include linear decomposition in a diversity of vector spaces and machine learning algorithms [15], [16], [18]. In this way, accurate control of bio-mimetic effectors is achieved for a finite number of specific tasks, such as grasping or cursor moving.

Our current understanding of the biophysics of the avian vocal organ, particularly our capacity to identify the dynamical mechanisms by which complex behavior occurs when the peripheral systems are driven by low dimensional, smooth instructions, allows us to propose an example of a different kind of bio-prosthetic solution. The model predicts a diversity of qualitatively different solutions to the system for continuous paths in a parameter space. Not only is this parameter space suggested by the model, but it is also physiologically pertinent.

We present a device that is driven by a freely behaving Zebra finch to produce realistic, synthetic vocalizations in real-time. The device is based on the real time integration of the mathematical model of the vocal organ on a Digital Signal Processor (DSP). It is controlled by the bird's subsyringeal air sac pressure gesture, which is transduced, digitized and fed to the DSP to provide the model with the appropriate path in parameter space.

The work is organized as follows. In the Methods section we describe the Zebra finch vocal organ and the mathematical model that accounts for its dynamics. We also discuss its applicability to the construction of a model-based bio-prosthetic device, and introduce the physiological motor gestures that relate to parameters of the model. We present the steps leading to the real-time implementation of the model on a device controlled by a spontaneously singing bird. In the Results section we show the example of a successful case. Finally, we summarize the results and discuss the impact of this device as an example of a kind of bio-prosthetic device enabled by the low-dimensional dynamical model of the peripheral effector.

## Methods

### Ethics statement

All experiments were conducted in accordance with the Institutional Animal Care and Use Committee of the University of Utah.

### Model for the vocal organ

One of the most studied species of songbirds is the Zebra finch. Its song presents a set of diverse acoustic features, which can be accounted for by the dynamics displayed by the mathematical model of its vocal organ [5], [11]. This low-dimensional mathematical model, when driven by the appropriate gesture in parameter space, is capable of producing realistic, synthetic birdsong. By implementing this model on a Digital Signal Processor, we are able to construct a bio-prosthetic vocal organ.

#### The Zebra finch vocal organ

The song of an adult Zebra finch is composed of the repetition of a highly stereotyped sequence of syllables, preceded by a variable number of introductory notes. A typical sequence or *motif* is made up of 2 to 8 distinct syllables [1], [2]. Vocalizations present a wide range of acoustic features, which include both tonal sounds where energy is concentrated in the fundamental frequency and spectrally rich ones, where energy is distributed among its harmonics.

**Figure 1:**
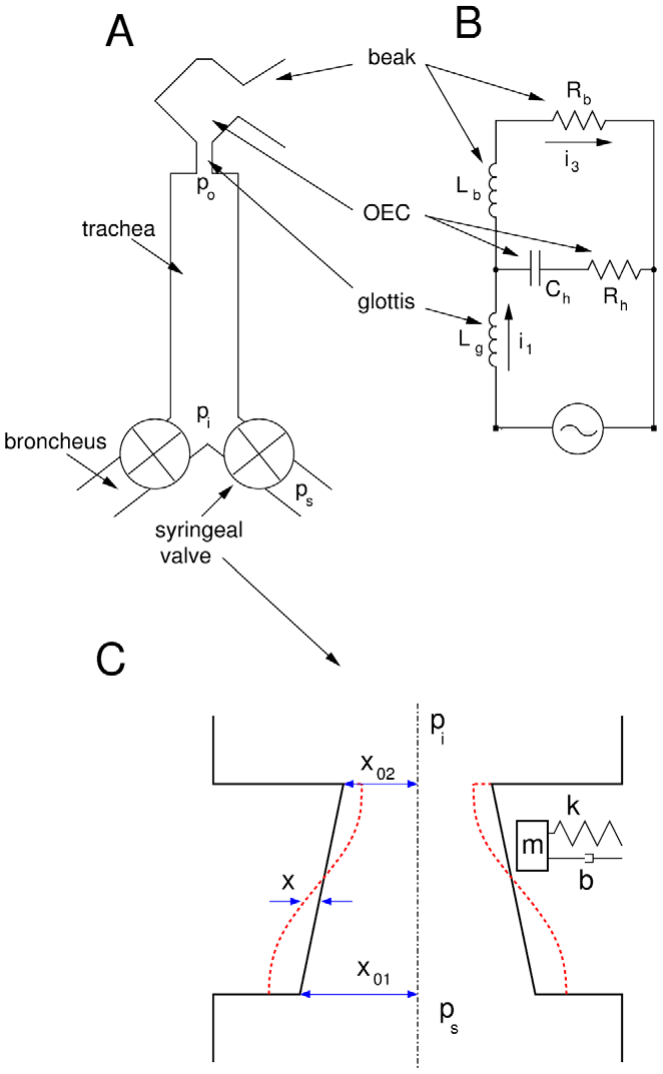
Sketch of the Zebra finch vocal system (A). Sounds are produced in the syringeal valves, and then filtered through the vocal tract. In the syrinx, the labia oscillate modulating the airflow. They support two coordinated modes of oscillation: an upward propagating superficial wave, and an oscillation around their mass centers (C). In the vocal tract we highlight trachea, glottis, OEC and beak. Pressure 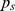 is the subsyringeal pressure, 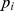 stands for the pressure at the input of the tube and 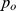 stands for the pressure at the output of the trachea. In order to compute the model, we write the equivalent circuit of the post-tracheal part of the vocal tract (B).

The oscine vocal system has two independent sound sources in the syrinx, where airflow is modulated to produce sound [2], [4]. The sounds produced in it travel then through the upper vocal tract: an acoustic pathway that introduces modifications of their acoustic features [1]. This is pictured in Fig. 1.

The bipartite syrinx of the oscines is a pair of valves, located at the junction of the bronchi and the trachea (see Figs. 1a and 1c). In each valve, small connective tissue pads, or labia, oscillate for high enough values of the airflow. Songbirds use their labia to produce sound in a similar way humans use vocal folds to produce speech. During vocalization, respiratory airflow sets the labia in an oscillatory regime. The oscillating labia modulate the airflow, resulting in a sound wave that then travels through the vocal tract. The airflow is controlled by the bird via the subsyringeal air sac pressure [11], [19]. The correlation of the electromyographic (EMG) activity of the ventral syringeal muscle (vS) and the fundamental frequency of a vocalization suggests that they control the tension of the oscillating labia [11], [20].

The upper vocal tract consists of the air-filled passages that link the syrinx to the environment. It determines much of the distribution of energy of the sound across its harmonic frequency components, defining a perceptual property as important as the timbre [21]–[23]. Its filtering characteristics depend on the acoustic properties of its components. Assuming that the compartments composing the upper vocal tract do not interact acoustically, they are considered individually. The most relevant components are the trachea, the glottis and oropharyngeal-esophageal cavity, and the beak [22], [24].

#### Model for the source

A model to account for the mechanism of sound production in the bird's syrinx that presents a good compromise between level of description and computational complexity was presented in [4], [11]. It is based on Titze's flapping mechanism, one of the simplest models to account for the transfer of the kinetic energy of airflow to vocal fold oscillations in humans [12]. Based on experimental observations, it assumes that the soft pieces of tissue or labia support two modes of vibration: an upward propagating wave and a lateral displacement around their midpoint positions. It has been suggested by videography of the folds during phonation [12], [19], [25] that these modes are coordinated in a way that energy is gained from the airflow in each cycle, making sustained oscillations possible.

The kinematic description of this mechanism is carried in terms of the displacement from equilibrium of the midpoint position of each labium 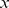. We assume that the profile of the syringeal valve is trapezoidal, as shown in Fig. 1c. The motion of the midpoint position of the labia will obey Newton's second law:
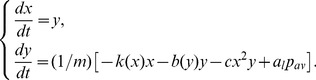
(1)In the right hand side of the second equation, the first term describes the nonlinear elastic restitution of the labium (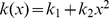), the second term represents nonlinear dissipation (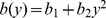), and the third term a nonlinear saturation that bounds the labial motion. The system is driven by the last term, which accounts for the force exerted by the inter-labial pressure (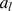 stands for the area of the labium).

In the driving term, 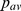 is the spatial average of the syringeal driving pressure. It is a function of the geometry of the folds that depends on the sub-syringeal pressure 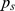, and the ratio of the tracheal and bronchial sections of the labial valve. Since the contribution of the pressure at the input of the tract 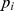 to the trans-syringeal pressure is much smaller than 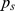, it is neglected [5]. This means that the sound source and the vocal tract are not considered to interact acoustically. The coordination of the two oscillation modes supported by the tissue, experimentally observed as a phase difference between the upper and lower extremes of the labia during phonation [19], [25], is taken into account in this function. By introducing a phenomenological parameter 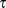 that describes the time it takes the wave propagating upward in the labia to cover half its length, the bronchial and tracheal areas of the valve (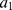 and 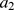) get to be written in terms of this time constant, the midpoint displacement of the labia and the resting (pre-phonatory) positions of the edges of the labia (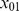 and 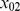) [12]. Finally, a phenomenologically corrected version of the Bernoulli equation is used to write the average syringeal pressure in terms of the ratio of the sections and the sub-syringeal pressure [11], [12]:
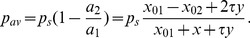
(2)Putting equations (1) and (2) together, an extended “flapping” model ruling the dynamics of the labia in the Zebra finch syrinx is complete.

In this model, acoustic features of the solutions are determined by physiologically meaningful parameters. Assuming that the coefficients in the restitution term of system (1) are proportional to the tension of the ventral syringeal muscles (vS), this model is capable of producing synthetic, realistic birdsong. The rationale behind this assumption is that the contraction of these muscles handles the stiffness of the labia by stretching them [11]. In [13], EMG activity recordings from electrodes implanted in the vS muscles were used to obtain a time dependent parametrization of the restitution (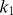), and air sac pressure data for 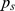. In this way, birdsong can be synthesized by driving the model with actual physiological data.

#### Model for the vocal tract

The oscillating labia in the syrinx modulate the airflow producing sound, which is modified as it goes through the vocal tract. Relevant acoustic features, such as the spectral content of vocalizations, are determined by the geometry of the vocal tract [5], [14], [26]. Important elements modulating birdsong in the vocal tract are the trachea, the oropharyngeal-esophageal cavity (OEC) and the beak, as noticed by Nowicki [21] and Fletcher *et. al.* [22], [24].

In order to produce realistic synthetic vocalizations, we introduce a model of the vocal tract as a dynamical system, which includes a tube approximating the trachea and a Helmholtz resonator to represent the OEC. The sound produced in the syrinx enters this system, and we are able to compute the sound radiated to the atmosphere.

The trachea, by its effect and its physiology, is approximated by a tube that is closed in the syringeal end and open at the glottis. The pressure at its input 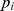 is determined by two main contributions: one originated from fluctuations originated in the syrinx, and the other caused by reflections at the open end. Considering both contributions, which are derived in [8], the pressure at the input of the trachea reads
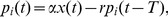
(3)where 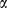 is proportional to the mean velocity of the flow, and 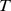 the time it takes a sound wave entering the tube to get to the open end and be partially reflected back with coefficient 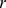.

The transmitted part of the pressure fluctuation 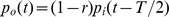 forces the air at the glottis, approximated by the neck of the Helmholtz resonator that stands for the OEC. The mass of air at the glottis, forced into the cavity, is subject to a restitution force exerted by the larger mass of air in it.

In acoustics, it is common to write an analog electronic computational model to describe a system of filters. The acoustic pressure is represented by an electric potential and the volume flow by the electric current [27]. In this framework, short constrictions are inductors, and cavities (smaller than the wavelengths) are well represented by capacitors. The equations for the equivalent circuit of the post-tracheal part of the vocal tract, shown in Fig. 1b, read:
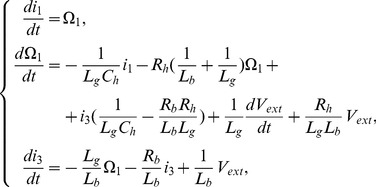
(4)where the electric components relate to geometric parameters of acoustic elements. Such relationships, which are standard in acoustics, can be found in [14], [27]. The pressure fluctuations at the glottal end of the trachea 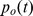 relate linearly to the electric tension 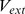 driving the circuit. Following the same scheme, the electrical potential at the resistor standing for the beak 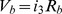 is the analogue of the pressure fluctuations at the output of the beak 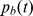. In our model, this quantity is the sound radiated by the vocal organ.

#### Dynamical analysis of the model

The mathematical model of the vocal organ represented by equations (1, 2, 3 and 4), rather than an attempt to obtain a statistical a filter or a set of causal rules between a coded motor command and a behavior, is a physical description of the complex peripheral effector. By studying the nonlinear dynamics of this model, specially that of its oscillatory solutions, we find nontrivial relationships between paths in parameter space and acoustic characteristics of sounds synthesized by it.

In this model, the non-interacting vocal tract acts as a passive filter; sounds produced in the syrinx are altered by it only on the relative weight of their harmonic components. This is why the dynamical analysis searching for qualitatively different oscillatory solutions is done on the equations ruling the dynamics of the syrinx. A thorough description of the set of solutions and bifurcations of the system (1,2) was carried out in [5], [28], and a phase portrait was constructed for the 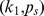 parameter space. This set is the most plausible to determine acoustic properties of sounds, since it relates to the restitution constant of the oscillating labia and the intensity of the force exerted by the airflow. These parameters are also identified with physiological instructions used by the bird for motor control [2], [11], [29].

**Figure 2:**
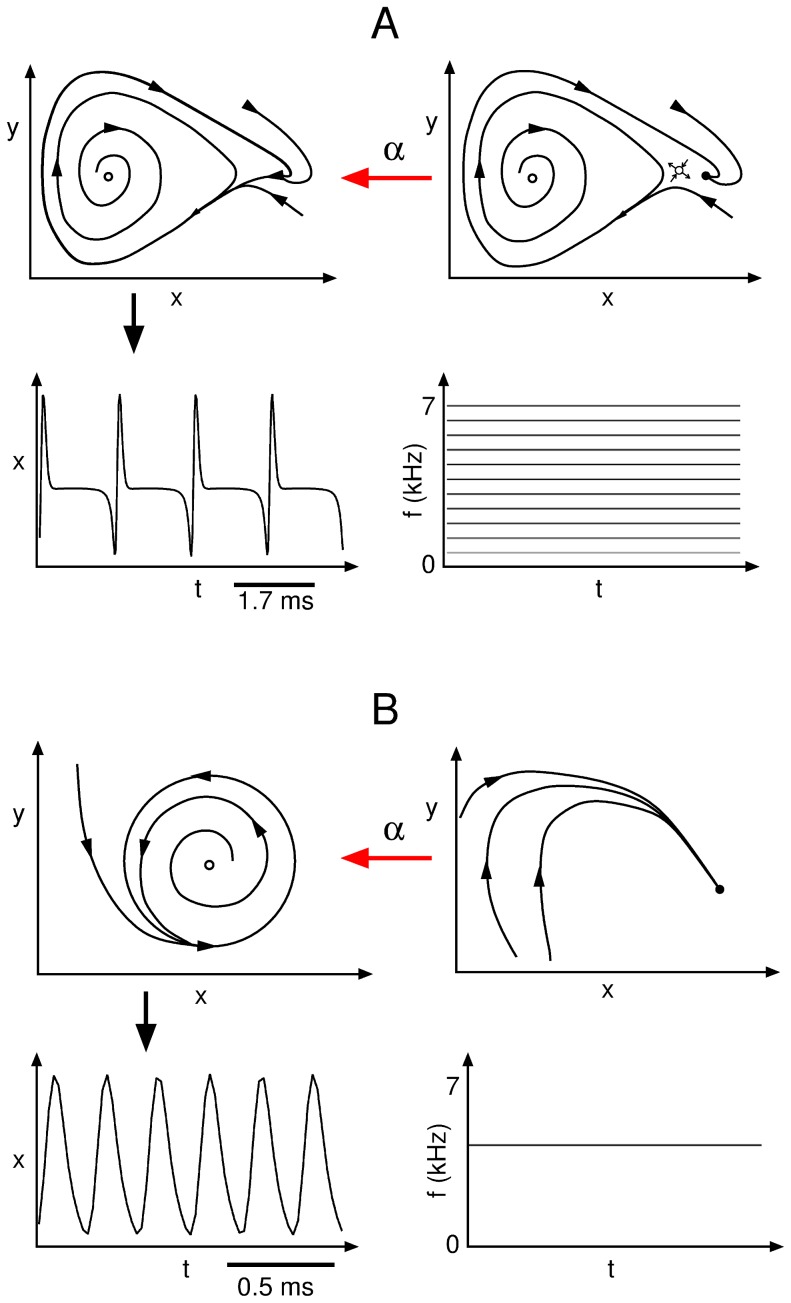
Sketch of the bifurcations found in the model of the syrinx leading to oscillatory solutions. When parameter 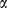 crosses a SNIC bifurcation, two fixed points collide within a limit cycle (A). The limit cycle is born with large period and rich spectral content, as sketched for the temporal series of the 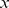 variable and its corresponding spectrogram. When parameter 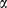 crosses a Hopf bifurcation, a stable fixed point loses stability against a newborn limit cycle (B). Close to the bifurcation, this limit cycle exhibits small period and ideally tonal spectral content, as sketched for the temporal series of the 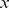 variable and its corresponding spectrogram. Both bifurcations occur at distinct regions in the 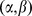 parameter space [5], [28].

Within the rich dynamical scenario displayed by the system, two distinct mechanisms giving rise to oscillatory solutions stand out: a Hopf and a Saddle Node on an Invariant Cycle (SNIC) bifurcations. They are sketched in Fig. 2, together with a schematic representation of the solutions and their spectral properties. Through a sub-critical Hopf bifurcation, one fixed point loses its stability and a small, stable limit cycle is created [30]. Oscillations are born with zero amplitude and finite frequency, therefore the sounds produced in this way are tonal. When a saddle and a non-saddle fixed point collide on a limit cycle, a SNIC bifurcation occurs at which oscillations with finite amplitude and infinite period are born [30]. Sounds produced by this mechanism have consequently low frequency and their spectral content is rich.

In addition to providing an orientation in the task of seeking the relevant control parameters, the dynamical analysis of the model makes way for the real-time implementation of the model by reducing its computational cost while retaining the relevant dynamics [13]. A polynomial coordinate transformation around a singular point in the parameter space is usually applied to dynamical systems to reduce the system to a normal form, leaving only the nonlinear terms needed to reproduce the set of bifurcations around that singularity [31]. Our model described by eqs. (1, 2) presents a Takens-Bogdanov singularity, where the SNIC and the Hopf bifurcation lines meet tangentially. The reduction to the normal form around that point eliminates many nonlinear terms and takes the system to a form where computation is much cheaper. When doing so, it retains the bifurcations that lead to oscillatory behavior. The new system reads:
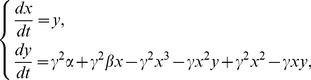
(5)where a proper mapping of air sac pressure and tension into the unfolding parameters 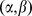 allows us to recover the qualitative dynamics. In [13] the relationship was explicitly computed; the basic operations involved are: a translation of the values of 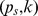 where a Takens Bogdanov bifurcation takes place in the physical model to 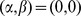, a multiplicative scaling, and a rotation of 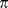.

### On-line synthesis driven by a freely behaving bird

The mathematical model for the vocal organ, the reduction of the system ruling the dynamics of the sound source, and the identification of the pertinent parameters accounting for its motor control, they all make way for the construction of a bio-prosthetic device. The parameters determining acoustic properties of vocalizations in the normal form (5) are physiologically meaningful and the set of differential equations is easy enough to compute in a portable platform such as a Digital Signal Processor. By fitting the parameters and integrating the system in real-time, synthetic song can be produced in a device controlled by the motor instructions elicited by a freely behaving bird.

In many bio-prosthetic solutions, the physiological motor gestures used to drive the device are degraded respect to those recorded in the intact subjects [15]. One application of this device is the performance of altered auditory feedback experiments [32]–[34]. In experiments enabled by this device, the bird's own auditory feedback can be replaced by synthetic birdsong computed in real-time. Since the synthetic feedback is produced by the integration of the model when fed with actual physiological motor gestures elicited by a freely behaving bird, alterations of the feedback are possible that are consistent with alterations in the motor gestures intended to produce them. Here, the prosthetic vocal organ is driven by a bird that is muted via the insertion of a cannula through its inter-clavicular air sac. Phonation is prevented as the airflow is bypassed away from the syrinx. As a side effect, the pressure pattern registered on the muted bird differs from that recorded in the intact animal.

The device reconstructs the intended motor gesture from this degraded pressure gesture to trigger integration of the model. When the pressure pattern corresponding to the syllables comprising a motif are identified, the mathematical model for the vocal organ is computed with the appropriate paths in parameter space, to produce the corresponding synthetic output.

#### Implementation

The electronic syrinx is capable of reproducing synthetic birdsong in real-time when driven by the air sac pressure of a freely behaving, muted bird. The pressure gesture is recorded, together with the bird's song, in order to fit the parameters of the model. The bird is then muted via a bypass of airflow away from the syrinx and its pressure gesture is digitized and fed to the Digital Signal Processor (DSP), where the model is implemented. An algorithm that reconstructs the pressure gesture of the intact bird is also implemented, to trigger the integration of the model on when the gesture corresponding to the first syllable of a motif is detected and off when it corresponds. Below there is a description of the procedure.

#### Record of the pressure gesture

The bird is cannulated. A cannula (*Silastic laboratory tubing*, 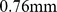 I.D., 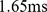 O.D.) is inserted through an incision in the thoracic air sac. The other end of the cannula is connected to a *Fujikura FPMC-07PGR* piezoelectric transducer, amplified and digitized and recorded at 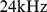, simultaneously with the sound (recorded with a *Takstar SGC568 microphone*) produced by the bird. The bird is placed in an acoustic box and sound and pressure are recorded while it spontaneously sings.

#### Reconstruction of the motor gesture

These data are then used to fit the physiologically meaningful parameters of the vocal organ model in its normal form (system (5)): the time-varying (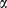, 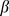) series are constructed so that the synthetic sound produced upon integration of the model matches the fundamental frequency and spectral content of the recorded song.

The characteristics of sounds produced by integrating the normal form of the model are determined by paths in the 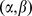 parameter space, which are transformations of the subsyringeal pressure and the activity of the ventral syringeal muscle [13], [14]. In order to produce synthetic vocalizations, the motor gesture is reconstructed from the recorded sound, looking for the excursions 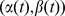 that lead to solutions of matching properties. The fitting procedure and the reconstruction of the motor gesture from the recorded song are detailed in [14].

Two important features that we seek to match are the spectral richness and the fundamental frequency of the vocalizations. One way of quantifying the spectral richness is to compute the Spectral Content Index (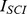) of a sound segment [5]. It measures how spread is the energy of a sound relative to its fundamental frequency, being its minimum 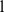 for perfectly tonal sounds.

Parameter 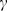 in system (5) determines the temporal scale. With this and the parameters in the description of the vocal tract fixed, a fundamental frequency and SCI 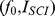 corresponds to every point in the 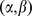 space. The dependence of the sound properties on the parameters is understood in terms of the distance, in parameter space, from the lines of bifurcation of the model. The further a point is from the Hopf and the SNIC bifurcation, the higher its corresponding fundamental frequency and spectral content index [5], [28].

The optimal value of the scaling factor 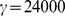 was found minimizing the accumulated distance in the acoustic features space 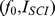 between 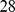 recorded segments (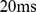 long) of birdsong and their targeting syntheses (see details in [14]). For every value of 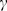 within a range, sounds corresponding to a grid of points in 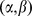 space were synthesized, and their fundamental frequency and spectral content index were computed. For each recorded segment, the synthetic sound minimizing the distance in 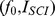 space was found. The sum of these minimum distances was carried over all the recorded segments, for every 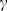 in the range. The value of 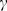 for which this overall distance is smallest is the optimal.

Setting also the parameters of the tract so that the resonance of the OEC lies close to 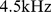 and the tube representing the trachea is 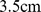 long, we reconstruct the motor gesture that synthesizes the bird's motif. To do so, the recorded song (sampled at 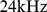) was decomposed into a sequence of 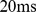 segments. For each segment, the SCI and fundamental frequency were computed. A search in the parameter space 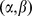 was performed until values were found which allow to synthesize sounds with the most similar acoustic features possible for the available parameter range. To do so, the fundamental frequency was computed for a grid in the parameter space, and the set of pairs 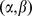 that produced synthetic sounds the closest to the fundamental frequency of the recording was selected. Within that set, the value of 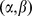 minimizing the distance in SCI value was chosen.

**Figure 3:**
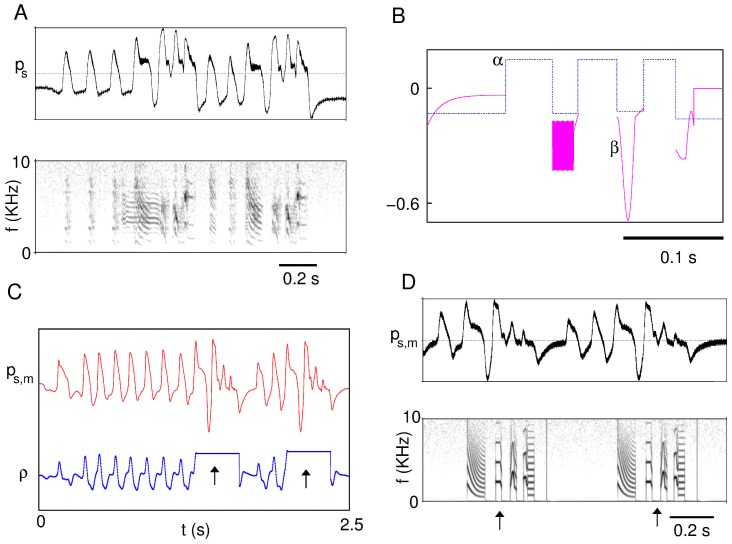
Illustration of the parameter fitting and calibration procedure. The thoracic air sac pressure is recorded together with the song; the pressure and corresponding sound of a bout are shown in (A). With these records, the temporal series 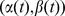 that originate the syllables corresponding to the motif are constructed (B). After muting the bird and registering the pressure gesture as it attempts to produce a motif, the detection algorithm is tuned. In (C) we show the degraded pressure gesture, together with the correlation with the chosen segment of the intact pressure gesture. The segments pointed out by arrows indicate the detection of the intention to sing a motif. Song is synthesized during these periods by integration of the model with the parameters 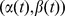 found previously. In (D), we show the pressure gesture of the muted bird and the output of the trigger/integrate algorithm.

By these means and upon smoothing to interpolate the values of the parameters within each segment, a table of values of the reconstructed motor gestures is obtained for every segment of the bird's motif. When the model is driven by them, synthetic song comparable to the one that was recorded is produced. An example of the fitted parameter series is illustrated in Fig. 3B, along with the actual song (Fig. 3A) and the motif synthesized by the model (Fig. 3D).

In [14], values of 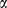 and 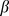 reconstructed in this way were used to estimate the values of air sac pressure and activity of the ventral syringeal muscle (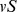), through a change of sign [13] and multiplication by a scaling factor. When the reconstructed gestures were compared against the actual, recorded values, the agreement was strong.

#### Muting of the bird

The bird is then muted via the insertion of an open cannula (*Braintree Scientific* MRE 05, 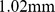 I.D., 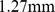 O.D.) through the interclavicular air sac. Airflow is deviated away from the syringeal valve, and phonation is prevented. Muting achieved in this way introduces as a side effect a degradation of the registered pressure, in a degree that is not systematic (for example, it is not constant in time for each subject, it is not an obvious algebraic transformation of the intact pressure gesture and its similarity with it varies from bird to bird).

The pressure pattern of the muted bird is then recorded as the bird attempts to produce song by producing the gesture corresponding to calls, introductory notes, and motifs.

#### Control of the model via the degraded pressure gesture

The recordings of the intact and degraded pressure gestures (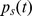 and 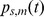 respectively) are used to tune the algorithm that will calculate the correlation of the muted pressure gesture with the intact pressure gesture to recognize the beginning of the first syllable of a motif. A test segment of length 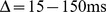 of the pressure gesture of the intact bird is selected, which contains the last part of the preceding introductory note and the first part of the first syllable of the motif. For each sample of the altered pressure gesture, a window of length 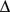 (ending at that point) is taken and its cross-correlation 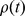 with the test segment of the intact gesture is computed.

The length 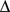 of the test segment of the intact pressure gesture is kept fixed, once determined. To determine it, we do an off-line exploration to select the segment that best enables discrimination of the beginning of the song bout, while minimizing the delay in the detection. The test segment should include as few samples of the first part of the motif as possible. The reason for this is that, when dealing with the stream of data in real-time, those samples required for the detection introduce a delay between the moment the bird attempts to sing and the beginning of the synthesis. In this exploration we seek the segment that complies with this condition and that can be distinguished from the calls and the introductory notes. To do so, we observe about 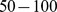 calls and introductory notes, and 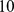 motifs. We look for the test segment for which the mean cross-correlation at the beginning of a motif is at least 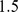 times the mean cross-correlation at calls and introductory notes.

This ensures that a correlation threshold can be set that leaves high probability of detection of the bout with low probability of false triggering (false inference of the onset of a motif from a call or any other note). Assuming that the cross-correlation values in the calls and introductory notes and the cross-correlation values in the beginning of a motif follow normal distributions with distinct mean and variance, these probabilities can be estimated. In the example shown here, the pressure gesture of a muted bird was recorded as it attempted to elicit 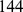 calls or introductory notes and 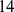 motifs. With the selected test segment, the mean cross-correlation for introductory notes and calls was slightly higher than 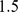 times the mean cross-correlation for the motifs. In this case, a threshold could be set for which the estimated probability of missing the trigger (computed as the area of the distribution of the correlation for the motifs to the left of the threshold) was close to 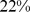. At the same time, the probability of false triggering (computed as the area of the distribution of the correlation for the calls and introductory notes to the right of the threshold) was close to 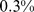.

The criterion for the selection of these quantities responds to the times of the procedure and the rate of success. The number of motifs used for calibration is the data available after one recording session of 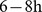, which takes place the day after the bird is muted. The proposed correlation threshold allows for the detection of the motif in most cases, while almost completely avoiding false triggering.

In Fig. 3C, we show the cross-correlation along a segment of the pressure gesture of a muted bird. The segment includes two bouts, each made of one motif starting after a different number of introductory notes. When the correlation threshold is reached, integration of the model is triggered, as shown in Fig. 3D. In this example (bird R01), the test segment of the intact pressure gesture included only the first 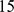 ms of the motif.

We also tune an algorithm that detects the interruption of the song, comparing properties of the muted pressure gesture with thresholds on absolute value and variation of the intact gesture during the subsequent motif. The song bout of a Zebra finch is highly stereotyped. Once a motif starts, a fixed sequence of syllables is followed, and a bout consists of the repetition of this motif, with the eventual interleaving of an introductory note between one and the next [1]. In order to control the electronic syrinx, it suffices to infer when a motif starts and when it is interrupted. Until the interruption is detected, the adequate vocalization is synthesized by integrating the model with the corresponding sequence of parameter values. The sequence is followed in segments of 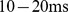, checking at the end of each window if integration of the next segment or silence corresponds. The end of phonation occurs when the pressure gesture remains below a certain threshold after registering a descending slope.

The method used to trigger the integration of the model introduces a delay. A segment of the first syllable is used to detect the onset of a motif in the muted bird's pressure gesture. The length of this segment determines the time it takes the synthesis to begin after the bird has attempted to produce the syllable. The synthesis, which begins after the detection, produces the song that corresponds immediately after that segment. In this way, the delay does not introduce a shift in time in the feedback. Instead, the part of the motif used for detection is skipped.

**Figure 4:**
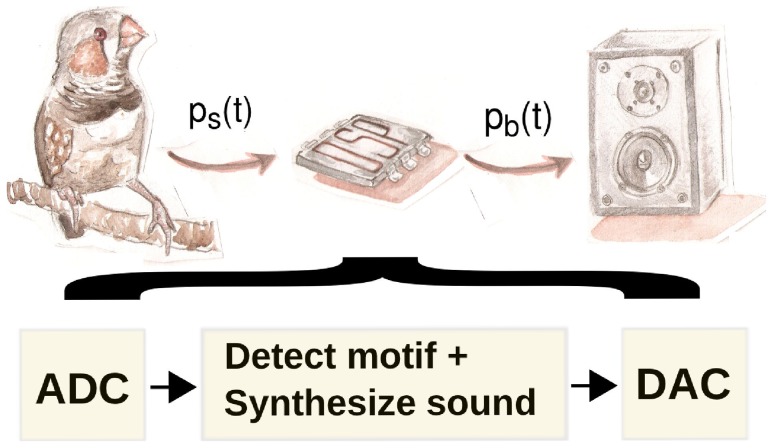
Sketch of the experimental setup where the muted bird drives the electronic vocal organ. The pressure gesture of the bird is recorded simultaneously with its song. Then, the parameters driving the normal form to produce synthetic song are reconstructed, and the bird is muted. The bird is then connected to the electronic syrinx via its thoracic air sac pressure, which is digitized and fed to the DSP. In the DSP, an algorithm detects the onset of the first syllable of the motif in the degraded gesture of the muted bird. Upon detection, the model is integrated in real-time while the attempt of the bird to continue with the motif is inferred from the motor gesture. The computed pressure fluctuations at the output of the beak are converted to an analog signal and played through a speaker located 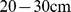 away from the bird.

The model with the constructed parameter paths (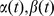) and the detection algorithms are then programmed in a DSP developing platform (*Texas Instruments DSK6713*). This platform contains a memory, a set of AD/DA converters for input and output and a floating point DSP running at 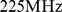. The muted bird is placed in the acoustic box and its pressure gesture is registered and fed to the DSP, where the detection algorithm runs in real-time. The recorded pressure gesture, sampled at 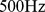, goes through the detection algorithm. When the beginning of the motif is inferred from the muted pressure gesture, the model is numerically integrated with the corresponding values of the parameters. Numerical integration of the model is carried out via the Euler method at 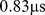 steps, in a way that output can be generated at 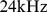. This output, corresponding to the sound produced by the syrinx, is used to compute the pressure at the output of the trachea and sent to another DSP that computes the dynamical equations that account for the vocal tract. The synthetic sound produced by these means is then converted into an analog signal, amplified and played through a speaker, placed in the acoustic box, about 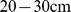 from the bird. The setup is illustrated in Fig. 4.

The sound registered in the box via the microphone, the direct analog output of the DSP system and the altered pressure gesture are digitized and recorded at 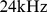.

## Results

### Subject-driven online synthesis

**Figure 5:**
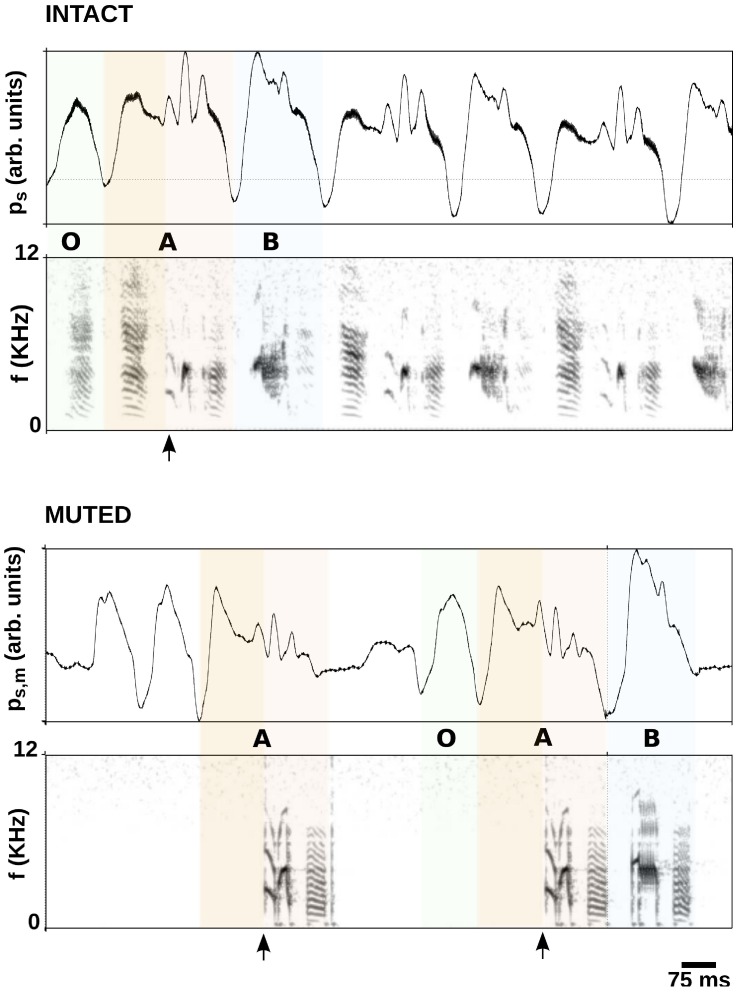
Intact song and subject-driven, synthetic song. Intact pressure gesture and sonogram, with different colors and opacity of shading indicating the different syllables, and an arrow indicating the segment of the first syllable used for detection (upper panels). When the muted bird drives the syrinx, we see in the sonogram that synthetic sound is produced after the first syllable is detected and until recognition of the interruption of the motif (lower panels).

The device succeeds in synthesizing song online when driven by the pressure gesture of a muted bird. From the altered motor gesture, the algorithm infers the segment of a motif intended by the bird and computes the model to produce the vocalizations. An example is illustrated in Fig. 5.

The upper panels of the figure display the recorded subsyringeal pressure and sonogram of a segment of a bout with its preceding introductory call. A song bout of this bird (B06) is composed of a number of introductory notes (O) and the repetition of a simple motif containing two syllables (A and B), indicated by different colors and opacity of shading in Fig. 5. An initial segment of syllable A (marked with clearer shading in the figure) is used to detect the intention to elicit a motif in the pressure gesture of the muted bird. The part that is produced upon triggering of the synthesis appears in a darker shade of the same color. These recordings were used to fit the parameters of the model to produce synthetic vocalizations showing a match in fundamental frequency and spectral content.

The bird is then muted and placed in the setup to drive the electronic vocal organ with its pressure gesture. In the lower panel of Fig. 5 we show the pressure gesture of the muted bird and the sonogram of the sound recorded by a microphone placed close (about 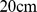) to the bird and the speaker. It can be noted in the sonogram that no sound is produced during the bird's attempts to phonate an introductory note. When the pressure motor gesture corresponds to the first syllable in the bout (syllable A), the instruction is recognized and the corresponding song is synthesized and played through the speaker. In the first bout, the bird only elicits the gesture corresponding to the first syllable, and then stops. In this case the algorithm detects the interruption and turns off the integration. In the second bout, the bird continues with the second syllable and drives the electronic syrinx to the end of the motif.

This example illustrates how this device works, and shows that it is successful in synthesizing the song motif as the bird drives it. We evaluate its success by counting the times the motif was properly detected and synthesized, and how many times a false trigger occurred. During a session of 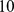 hours (the second day after the muting took place), a muted bird elicited about 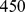 calls, out of which less than 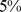 generated false triggering of synthetic song. In most cases the false trigger event was recognized by the algorithm and silenced after less than 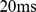. The rate of success in detecting the beginning of a bout was of about 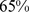 in 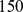 attempted bouts elicited by the bird.

Despite the variability of the altered pressure gesture in the subsequent days (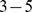 days after the muting), a brief calibration before each daily session allowed the rates of success and false triggers to be maintained. To do this calibration, the pressure gesture was recorded during 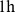 and these data were used to re-set the cross-correlation thresholds, while keeping the test segment of the intact pressure gesture previously selected.

### Perspectives for BMI

We show here that realistic vocal behavior is synthesized in real-time by our device, as it is controlled by the spontaneous behavior of a muted bird, a physiological signal (its air sac pressure) that is degraded in respect to the one recorded in the intact bird. The computing platform is a low cost, portable processor, and the initial rate of success is high. This is an encouraging example of the plausibility of a kind of interface between the central motor pattern generator and the synthetic, bio-mimetic behavior. DSP technology is being implemented in a variety of biologically inspired problems, and together with Field Programmable Gate Array technology (FPGA) is likely to become a standard solution for a variety of bio-mimetic applications [15], [35].

Brain computer and brain machine interfaces (BCI and BMI) typically read physiological data and attempt to decode motor instructions that drive peripheral devices in order to produce synthetic behavior [15], [17], [35], [36]. Because we have a physical model of the peripheral effector, the origins of the complexity of the behavior were linked to smooth paths in a low dimensional parameter space. Following the identification of the pertinent parameters and their physiological link to the pattern generator (activity of the ventral syringeal muscle and sub-syringeal air sac pressure), a further simplification of the system was carried out, on dynamical grounds, by eliminating irrelevant nonlinear terms (performing a reduction of the model to its normal form). This led to the possibility of implementing our bio-prosthetic device on a programmable electronic platform. Since the computing capabilities of the platform are greatly enhanced by the low costs of our implementation, technological advances in this front will have great impact on the complexity of the peripheral biomechanical system that can be emulated.

## Discussion

### Model-based bio-prosthesis

We have built a device that emulates complex motor behavior when driven by a subject by its actual (yet degraded) physiological motor gestures. It successfully reproduces the result of the stereotyped motor gesture that leads to the behavior, *i.e.*, the diverse and complex set of sounds comprising the bird's song bout. The realistic synthetic vocalizations are produced in real-time, by computing a mathematical model of the vocal organ on a portable Digital Signal Processor.

The relative computational and technological simplicity of the device relies on the current level of understanding of the peripheral biomechanical effector [1], [2], [12], [24]. We have been able to construct a physical model of the syrinx that presents the adequate level of description and converges to a low dimensional (as low as two dimensions) dynamical system. The deterministic model of the vocal organ on which our device is based is not a statistical attempt to capture causal relationships between motor commands and behavior; instead, it is a hierarchization of the interactions within the biomechanical periphery and with the pattern generator. It aims to identify the dynamical mechanisms by which the behavior is produced.

Furthermore, exploration of the model leads to the finding that much of the diversity and complexity of the behavior can be explained in terms of the dynamical features of this nonlinear system [5], [28], requiring only simple instructions of the nervous system to produce a rich variety of vocalizations. Just as it is identified that a low dimensional system reproduces the main features of the complexity of the vocal organ, it can also be concluded that the control parameters are few and their behavior is simple (*i.e.*, the physiological motor gestures linked to the paths in 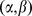 space are smooth). In this way, the parameter space of the model not only suggests the pertinent physiological instructions determining the main properties of the output but also how they are expected to behave. Paths in parameter space reconstructed in order for the model to produce vocalizations matching experimental recordings are indeed effective in predicting the physiological motor gestures [14].

In addition, knowledge of nonlinear dynamics allows us to find the simplest system with equivalent oscillatory behavior. The reduction of the low dimensional mathematical model for the syrinx to its normal form reduces the computational requirements and makes way for the implementation on a real-time computing solution, such as a DSP.

### Conclusion

Realistic vocal behavior is synthesized online, controlled by the motor gesture of a freely behaving muted bird, which is a physiological signal that is degraded respect to the one recorded in the intact bird. This was achieved by computing in real time a mathematical model describing the mechanisms of sound production in the interface between the motor pattern generator and the behavior, the highly nonlinear vocal organ. The computing platform is a low cost, portable processor. This successful avian vocal prosthesis is an encouraging example of the plausibility of a kind of interface between the central motor pattern generator and the synthetic, bio-mimetic behavior. An advance towards models in which certain complex features of the motor behavior are understood in terms of the underlying nonlinear mechanisms of the peripheral effectors has the potential to enhance solutions of brain-bio-mimetic effector interfaces in many ways.
